# Thiamine Concentration in Human Milk Is Correlated With Maternal and Infant Thiamine Status: A Cross‐Sectional Analysis of the Lao Thiamine Study

**DOI:** 10.1111/mcn.70027

**Published:** 2025-04-10

**Authors:** Sonja Y. Hess, Charles D. Arnold, Taryn J. Smith, Lindsay H. Allen, Daniela Hampel, Kerry S. Jones, Damon A. Parkington, Sarah R. Meadows, Dalaphone Sitthideth, Sengchanh Kounnavong

**Affiliations:** ^1^ Institute for Global Nutrition and Department of Nutrition University of California Davis Davis California USA; ^2^ Department of Women's and Children's Health Institute of Life Course and Medical Sciences, University of Liverpool Liverpool UK; ^3^ USDA‐Agricultural Research Service Western Human Nutrition Research Center Davis California USA; ^4^ Nutritional Biomarker Laboratory MRC Epidemiology Unit, University of Cambridge Cambridge UK; ^5^ Lao Tropical and Public Health Institute Vientiane Lao People's Democratic Republic

**Keywords:** breastmilk thiamine, erythrocyte transketolase, Laos, thiamine diphosphate, Vitamin B1

## Abstract

The human milk content of some micronutrients including thiamine depends on maternal status, and if low, breastfed infants are at risk of deficiency. Thiamine deficiency remains an important cause of morbidity and mortality among infants in Asia. We aimed to explore correlations between maternal thiamine diphosphate (ThDP) or erythrocyte transketolase activation coefficient (ETKac) and human milk thiamine concentration (MTh) and between MTh and infant ThDP or ETKac among breastfed infants < 6 months of age in northern Lao PDR. Hospitalized infants (aged ≥ 21 days) with symptoms suggestive of thiamine deficiency were eligible. Infants in a community comparison group were matched by age, sex and residence. Venous whole blood ThDP and MTh were determined by HPLC‐FLD, and ETKac in washed erythrocytes by UV spectrophotometry. Associations between biomarkers were assessed using Spearman's *ρ* correlations and linear regression. Among all women combined (*n* = 489), the prevalence of ThDP < 95 nmol/L was 78.5%, elevated ETKac (> 1.25) 52.6%, and low MTh (< 90 µg/L) 45.4%. Maternal ThDP was moderately correlated with MTh (*ρ* = 0.50) and ETKac was strongly correlated with MTh (*ρ* = −0.71). Among all infants combined (*n* = 359), the prevalence of ThDP < 95 nmol/L was 79.2% and elevated ETKac (> 1.25) 50.2%. MTh was moderately correlated with infant ThDP concentration (*ρ* = 0.39) and with infant ETKac (*ρ* = −0.52). Maternal thiamine status predicts the thiamine concentration in human milk, and thiamine status of breastfed infants < 6 months of age depends on the thiamine provided through this milk. Effective interventions are needed to improve maternal and infant thiamine status and wellbeing.

**Trial Registration:**

Clinicaltrials.gov NCT03626337.

## Summary

- Low thiamine status was prevalent among women and their breastfed infants < 6 months of age in northern Lao PDR.
- Among breastfeeding women, whole blood thiamine diphosphate concentration and erythrocyte transketolase activity coefficient were correlated with thiamine concentration in their milk.
- Among exclusively and predominantly breastfed infants < 6 months of age, thiamine concentration in their mothers' milk was correlated with both biomarkers of thiamine status.
- Maternal thiamine supplementation, ideally as part of a multiple micronutrient supplement, and/or increasing dietary diversity is urgently needed to improve thiamine status among women and their infants.

## Graphical abstract

Thiamine status of breastfed infants < 6 months of age is correlated with human milk thiamine concentration, which in turn is strongly correlated with maternal thiamine status in northern Lao PDR. Improving maternal thiamine status is important for the health and wellbeing of both mother and infant.

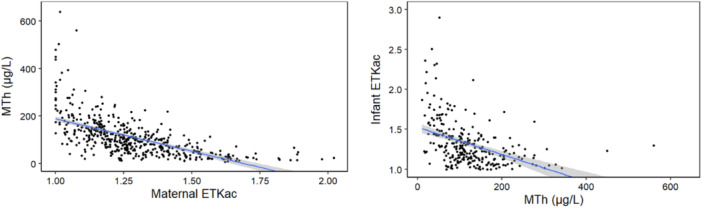

## 1 Introduction

There is strong evidence that exclusive breastfeeding for the first 6 months reduces the infant's risk of infectious morbidity and mortality (Victora et al. 2016). Because of its benefits for infants and mothers, exclusive breastfeeding for the first 6 months of life is recommended by the World Health Organization (2024). While human milk provides infants with essential nutrients and many protective antibodies, the milk content of several micronutrients depends on the maternal status and/or current intake (Allen 2012). For most water‐soluble micronutrients, maternal supplementation can increase the nutrient content in milk and help to support the adequate micronutrient status of the infant. Thiamine, also known as vitamin B_1_, is one of the micronutrients where maternal deficiency may result in low thiamine milk content (MTh). Indeed, in regions with a high prevalence of thiamine deficiency, breastfeeding has been recognized as one of the risk factors for clinical manifestations of thiamine deficiency among infants (World Health Organization 1999; Smith et al. 2021).

The risk of thiamine deficiency is elevated when thiamine requirements are increased during pregnancy and lactation (US Institute of Medicine 1998). Meeting these higher thiamine requirements is particularly challenging among populations relying on monotonous diets based on white rice or cassava (World Health Organization 1999). Two biomarkers are commonly used to determine thiamine status: thiamine diphosphate (ThDP) and erythrocyte transketolase (ETK) (Whitfield 2025). ThDP is the metabolically active form constituting of approximately 80% of total body thiamine and can be assessed in either whole blood or erythrocytes (Talwar et al. 2000). ThDP is a cofactor for many essential enzyme complexes, including transketolase. Erythrocyte transketolase (ETK) activity is considered a functional marker of thiamine adequacy (Jones et al. 2021). There is no international consensus on cutoffs of ThDP or ETK activity coefficient (ETKac), that should be used to define thiamine deficiency, but an ETKac cutoff > 1.25 is generally used to define high risk of thiamine deficiency (US Institute of Medicine 1998; Turck et al. 2016). In human milk, 200 μg thiamine/L has been suggested as adequate for the majority of infants in the first half‐year of life, and is recommended by the European Food Safety Authority (EFSA 2013).

This study is a secondary analysis of the Lao Thiamine Study, a prospective cohort study implemented in the northern Lao People's Democratic Republic (Lao PDR; Hess et al. 2020). The predictive model for thiamine‐responsive disorders among infants and young children has recently been published elsewhere (Smith et al. 2024). We also previously reported that none of the thiamine biomarkers (ThDP, ETKac, MTh) reliably identified individual children with thiamine‐responsive disorders (Hess et al. 2024). In the present study, we explore correlations between (1) maternal ThDP or ETKac and MTh and, (2) between MTh and infant ThDP or ETKac among breastfed infants < 6 months of age. Secondary objectives were to define a population‐specific cutoff for MTh corresponding to maternal ETKac > 1.25 among lactating women and to infant ETKac > 1.25 among exclusively and predominantly breastfed infants.

## 2 Methods

### 2.1 Study Site and Study Population

The present study was limited to cross‐sectional data collected at enrollment among breastfed infants < 6 months of age and their mothers. The data was derived from the Lao Thiamine Study implemented in Luang Prabang, northern Lao PDR, which enrolled a hospital cohort and a community comparison group, as previously described (Hess et al. 2020). Infants and young children 21 days to < 18 months of age were eligible for study enrollment if they were hospitalized at the Lao Friends Hospital for Children with at least one clinical sign or symptom suggestive of thiamine deficiency disorder (Supporting Information S1:Table S1). For the community comparison group, women‐infant dyads were frequency‐matched by residence, age and sex to the hospitalized children. To achieve a well‐balanced match and to avoid seasonal bias, the frequency matching was done on a weekly basis. The purpose of including a community group was to serve as a comparison group for evaluating thiamine deficiency in a non‐hospitalized cohort (Hess et al. 2020).

The study and consenting procedures received ethical approval by the National Ethics Committee for Health Research, Ministry of Health, Lao PDR (11/2019) and the Institutional Review Board of the University of California, Davis (1329444). Written informed consent was obtained from at least one parent or the primary caregiver for the children's participation, and from the women for their own participation. Data was collected from June 2019 to December 2020.

### 2.2 Data Collection

Mothers were asked structured survey questions about their socioeconomic status (SES), their own diet and health and their breastfeeding practices during the previous 24 h in the community, and in the hospital group during the 24 h before going to the hospital (FAO 2021; World Health Organization 2021). Food security was assessed using the Household Food Insecurity Access Scale (Coates et al. 2007). Anthropometric assessments were completed for women and children following recommended protocols (Cashin and Oot 2018). A physical exam was performed including neurological examination of women and infants. Women were asked to perform a heel‐ and a toe‐walk test. This was considered abnormal, if the woman could not walk 10 m while walking on her heels and maintaining full ankle dorsiflexion, or walking on her toes, respectively (Nilles et al. 2018). Women were also asked to rise from a squat without additional support and whether they experienced tingling in fingers or feet in the previous 14 days.

### 2.3 Blood Collection and Laboratory Analyses

Venous blood was collected from children into 6 mL BD EDTA vacutainer tubes (ref 367863, Becton, Dickinson & Company, Franklin Lakes, NJ, USA) and from women into 10 mL BD EDTA vacutainer tubes (ref 368589, Becton, Dickinson & Company, Franklin Lakes, NJ, USA). Blood samples were collected from mother–child dyads on the same day, and were processed within < 2 h in the hospital laboratory or a temporary mobile laboratory in the community. Whole blood was aliquoted into amber microcentrifuge tubes and stored at −80°C for ThDP assessment. The remaining blood was centrifuged at 3200 rpm (~1200) for 10 min (DM0412S, DLAB Scientific Co. Ltd., Beijing, China). After aliquoting plasma and removal of the buffy coat, packed erythrocytes were washed three times in 0.9% saline and centrifuged at 4000 rpm (~1900*g*) for 10 min each time. All samples aliquoted in the community were frozen in an electronic cool box at −20°C and transferred to the −80°C freezer at the hospital.

At the Nutritional Biomarker Laboratory (NBL) at the University of Cambridge (UK), ThDP was determined by the thiochrome reaction coupled with high‐performance liquid‐chromatography fluorescence detection (HPLC‐FLD) and was based on a modified version of a previously published method (Lu and Frank 2008). The assay of ETK activity was completed using an UV spectrophotometer before and after the addition of excessive exogenous ThDP (Jones et al. 2021). Two single donor, whole blood quality control (QC) samples with mean measured concentrations of 167 and 83 nmol/L, and a third in‐house QC material prepared by dilution of whole blood with phosphate‐buffered saline which had a concentration of 55 nmol/L were used to determine analytical imprecision for ThDP. Between‐run percent coefficient of variation (%CV) was ≤ 7%. In addition, Chromsystems whole blood calibration standard for vitamin B_1_ (Chromsystems, Munich, Germany) was measured and had a between‐run %CV of 8%. Three QC samples, prepared from single donor samples and stored at −70°C as erythrocyte lysates were measured with each batch for ETKac. Due to the difficulty of obtaining blood samples in sufficient quantity from people with high ETKac in the UK, all QCs had ETKac values < 1.2; between‐run %CVs were ≤ 3%.

### 2.4 Human Milk Collection and Laboratory Analyses

We collected a milk sample on the same day as the venous blood sample was collected. After cleaning the breast with a sanitary wipe (Hygea Obstetrical Towelette, PDI Healthcare, Orangeburg, NY, USA), milk from the fuller breast was collected with an electronic hospital‐grade breast pump (Symphony pump, Medela AG, Baar, Switzerland) until the breast was empty. We collected human milk any time of day because the circadian variation in thiamine concentration is small as previously reported by Hampel et al. (2017), but time of day, time since last meal, breast side, time since last emptying of that side, and milk volume were recorded. Samples were mixed well before aliquoting into 1.5 mL amber microcentrifuge tubes and frozen at −80°C until analysis at the USDA, ARS Western Human Nutrition Research Center (Davis, CA, USA). Samples were analyzed by HPLC‐FLD after derivatization of the thiamine vitamers to their trichome esters (Hampel et al. 2016). For quality control, an in‐house pooled human milk sample was used during the analysis (Hampel et al. 2012). MTh concentrations were calculated as: free thiamine + (thiamine monophosphate × 0.871) + (thiamine diphosphate × 0.707).

### 2.5 Statistical Analysis

All analyses were completed in Stata v16.1 (StataCorp LLC, College Station, TX) following a pre‐defined statistical analysis plan (Hess, Smith, Arnold 2024). To ensure we considered baseline thiamine status and did not include participants with biomarker concentrations artificially increased by recent thiamine administration, we excluded thiamine biomarkers for women and children who reported supplementation before the blood draw and/or had a free thiamine concentration greater than the 90th percentile of the study sample (> 202.7 nmol/L).

For the purpose of the present paper, we defined low ThDP as < 95 nmol/L, as previously suggested for adults (Schrijver et al. 1982; Ihara et al. 2005), and we applied the common threshold for high risk of thiamine deficiency as ETKac > 1.25 (Whitfield 2025). Based on recent data from the Mothers, Infants and Lactation Quality (MILQ) study (Allen et al. 2021), we defined low MTh total thiamine concentration as < 90 μg/L, which is the 25th percentile of values from the MILQ study (unpublished data, personal communication L.H. Allen).

The below described analyses were performed for all study participants combined, and explored by study group (i.e., hospital and community). ETKac, ThDP, and MTh were all right skewed and non‐normal by Shapiro–Wilk testing. Consequently, they were log‐transformed for regression analysis or non‐parametric methods were used as indicated. Differences in baseline characteristics and thiamine biomarkers between the hospital and community cohorts were assessed using Mann–Whitney *U* tests for continuous variables and *χ* ^2^ tests for categorical variables. Associations between maternal thiamine status indicators, human milk concentration and infant thiamine status indicators were assessed using Spearman's *ρ* correlations and linear regression for all participants combined. Following the flow of nutrients through the system, maternal status served as the predictor of human milk concentration and in separate models, human milk concentration served as the predictor for infant status. Bivariate unadjusted models were first fit to assess the observed association; then multivariable models were fit to assess the association while adjusting for potential confounding. Potential confounders included infant age, breastfeeding practice and other pre‐specified maternal, child, and household characteristic variables that were associated with the outcome in bivariate models with *p* < 0.1. To explore whether the indicated associations were modified by breastfeeding status or child's age, we introduced interaction terms with human milk concentration. If an interaction was marginally significant (*p* < 0.1), we further explored the interaction through visualization and stratified analyses. In addition, we also visualized the prevalence of low ThDP and high ETKac across MTh distribution quartiles and tested statistical differences using *χ* ^2^ tests.

We used an AUROC analysis framework with optimal cutoffs selected via the closest‐to‐(0.1) corner cut‐point approach to determine MTh cutoffs corresponding to ETKac > 1.25. These analyses were conducted separately for women and exclusively or predominately breastfed infants and were also repeated among subgroups based on cohort, exclusivity of breastfeeding, infant age, socioeconomic status, and food insecurity.

## 3 Results

### 3.1 Characteristics of Women and Their Children

**Figure 1:**
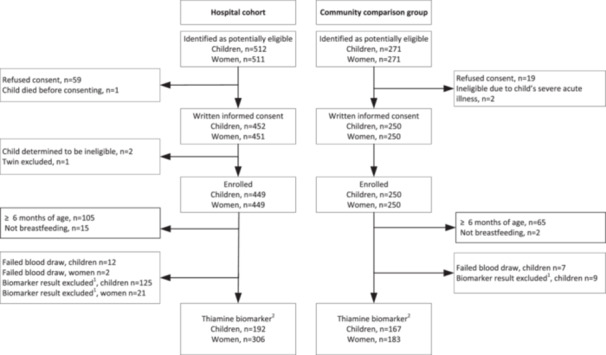
Flow chart of study participants. ^1^Women and children who reported supplementation before the blood draw and/or had a free thiamine concentration greater than the 90th percentile of the study sample were excluded. ^2^ Number of study participants with at least one thiamine biomarker result and/or human milk thiamine concentration. Number of mother‐infant dyads where either the women or the child was included: *n* = 319 in the hospital cohort, and *n* = 183 in the community comparison group.

For the main study, 512 children were identified as potentially eligible in the hospital cohort and 271 in the community. Of these, 449 and 250 dyads of mothers and children were enrolled in the hospital and community groups, respectively (Figure 1). After exclusion of dyads with infants and children older than 6 months, non‐breastfeeding women‐infant dyads, and women and infants who had free thiamine concentrations above the 90th percentile and/or because they reportedly received thiamine before their blood draws, 306 women and 192 infants provided at least one thiamine biomarker result in the hospital cohort (319 dyads with biomarker from either woman or child) and 183 women and 167 infants in the community (183 dyads with biomarker from either woman or child) for the present analyses.

The mean age of women was 24.5 ± 6.5 years and for infants it was 2.7 ± 1.4 months (Table 1). Even though participants in the community comparison group were frequency matched by location of residence and age and sex of the child, there were significant differences in ethnic identification. Specifically, in the hospital group, the majority of women belonged to the Hmong ethnic group (61.2%), while in the community 25.1% of women identified as Hmong, and 48.1% as Khmu. The prevalence of moderate to severe food insecurity was significantly higher in the hospital compared to the community group (40.5% vs. 24.6%, *p* < 0.001). More women in the hospital cohort reported tingling in fingers and toes in previous 2 weeks, which may indicate that more women in the hospital cohort suffered from thiamine deficiency compared to the community comparison group. As previously reported in more detail (Smith, Tan, et al. 2022), at the time of interview, 41.0% of women reported that they presently and 52.0% that they previously followed a restricted postpartum diet for traditional and cultural reasons, with no significant difference between the hospital and the community mothers. The percent of women who achieved the minimum dietary diversity was low, but significantly lower in the hospital compared to the community (8.7% vs. 15.3%, *p* = 0.023). Breastfeeding practices differed by setting (*p* < 0.001) with the vast majority of women reporting that they exclusively breastfed their infant at the time of data collection (81.5% in hospital vs. 79.8% in community). More women in the hospital reported predominant breastfeeding compared with the community group (7.8% vs. 3.3%) and mixed milk feeding (9.4% vs. 6.0%). In contrast, more women in the community reported having already introduced complementary food to their infant < 6 months of age while continuing breastfeeding (10.7%) compared to women in the hospital (1.3%).

**Table 1:** Baseline characteristics of women and their infants < 6 months of age in the hospital cohort and community comparison group of the Lao Thiamine Study, among participants where child and/or maternal thiamine biomarkers were available.

Characteristics	All	Hospital	Community	*p*‐value
N mother‐child dyads^a^	502	319	183	
*Maternal characteristics*				
Age (y)	24.5 (6.2)	24.5 (6.3)	24.6 (6.1)	0.705
Pregnant, *n* (%)	7 (1.4)	4 (1.3)	3 (1.6)	0.723
Province of residence, *n* (%)				0.290
Luang Prabang	463 (92.2)	298 (93.4)	165 (90.2)	
Oudomxay	28 (5.6)	14 (4.4)	14 (7.7)	
Xayaboury	9 (1.8)	5 (1.6)	4 (2.2)	
Other	2 (0.4)	2 (0.6)	0 (0.0)	
Ethnic group, *n* (%)				< 0.001
Lao	75 (15.2)	35 (11.2)	40 (21.9)	
Khmu	170 (34.3)	82 (26.3)	88 (48.1)	
Hmong	237 (17.9)	191 (61.2)	46 (25.1)	
Other	13 (2.6)	4 (1.3)	9 (4.9)	
Education, *n* (%)				< 0.001
No formal education	95 (19.2)	74 (23.7)	21 (11.5)	
Some/completed primary	159 (32.1)	96 (30.8)	63 (34.4)	
Some/completed secondary	209 (42.2)	130 (41.7)	79 (43.2)	
College/university	32 (6.5)	12 (3.9)	20 (10.9)	
Occupation, *n* (%)				< 0.001
Does not work	9 (1.8)	4 (1.3)	5 (2.7)	
Housewife	106 (21.4)	51 (16.4)	55 (30.1)	
Farmer	317 (64.0)	232 (74.4)	85 (46.5)	
Unskilled labourer	1 (0.2)	1 (0.3)	0 (0.0)	
Skilled worker^b^	62 (12.5)	24 (7.7)	38 (20.8)	
Household SES index^c^	−0.04 (1.00)	−0.17 (0.95)	0.18 (1.05)	< 0.001
Food insecurity category, *n* (%)				< 0.001
None to mild	323 (65.4)	185 (59.5)	138 (75.4)	
Moderate to severe	171 (34.6)	126 (40.5)	45 (24.6)	
Gravidity	2.6 (1.9)	2.8 (2.1)	2.3 (1.5)	0.061
ANC visits, *n* (%)				< 0.001
0–3	161 (32.8)	126 (40.8)	35 (19.2)	
4–7	212 (43.2)	126 (40.8)	86 (47.3)	
≥ 8	118 (24.0)	57 (18.5)	61 (33.5)	
Reported prenatal supplementation, *n* (%)	421 (85.2)	251 (80.7)	170 (92.9)	< 0.001
Dietary diversity score	3.1 (1.3)	2.9 (1.3)	3.5 (1.3)	< 0.001
Achieved MDD‐W^d^, *n* (%)	55 (11.1)	27 (8.7)	28 (15.3)	0.023
Restricted diet postpartum, *n* (%)				0.978
Never restricted	35 (7.0)	22 (6.9)	13 (7.1)	
Previously restricted	261 (52.0)	165 (51.7)	96 (52.5)	
Presently restricting	206 (41.0)	132 (41.4)	74 (40.4)	
Weight (kg)	49.0 (6.9)	48.3 (6.6)	50.2 (7.2)	0.003
Height (cm)	149.6 (5.7)	148.7 (5.6)	151.1 (5.5)	< 0.001
BMI (kg/m^2^)	21.9 (2.7)	21.8 (2.6)	22.0 (2.9)	0.592
BMI < 18.5 kg/m^2^, *n* (%)	37 (7.6)	22 (7.3)	15 (8.3)	0.681
MUAC (cm)	24.2 (2.5)	24.1 (2.6)	24.3 (2.3)	0.161
Maternal neurological exam				
Lethargy, *n* (%)	137 (27.7)	112 (35.9)	25 (13.7)	< 0.001
Reduced appetite, *n* (%)	86 (17.4)	73 (23.4)	13 (7.1)	< 0.001
Reported tingling in fingers and toes in previous 2 weeks, *n* (%)	144 (29.1)	118 (37.8)	26 (14.2)	< 0.001
Heel walk abnormal, *n* (%)^e^	18 (13.2)	9 (10.3)	9 (18.4)	0.185
Toe walk abnormal, *n* (%)^e^	9 (6.6)	6 (6.9)	3 (6.1)	0.862
Rise from squat abnormal, *n* (%)^e^	2 (1.5)	1 (1.2)	1 (2.0)	0.678
*Infant characteristics*				
Age (month)	2.7 (1.4)	2.6 (1.4)	2.8 (1.5)	0.190
Male, *n* (%)	290 (57.9)	190 (59.6)	100 (55.0)	0.314
Breastfeeding status, *n* (%)				< 0.001
Exclusive breastfeeding	406 (80.9)	260 (81.5)	146 (79.8)	
Predominant breastfeeding^f^	31 (6.2)	25 (7.8)	6 (3.3)	
Mixed milk feeding^g^	41 (8.2)	30 (9.4)	11 (6.0)	
Continued breastfeeding^h^	24 (4.8)	4 (1.3)	20 (10.7)	
Length (cm)	57.0 (4.4)	56.4 (4.5)	58.0 (4.0)	< 0.001
Weight (kg)	5.1 (1.2)	5.0 (1.2)	5.4 (1.1)	< 0.001
MUAC (cm)	12.5 (1.4)	12.3 (1.5)	13.0 (1.0)	< 0.001
Head circumference (cm)	38.5 (2.3)	38.2 (2.3)	38.8 (2.2)	0.003
Length‐for‐age z‐score	−1.15 (1.30)	−1.35 (1.42)	−0.82 (0.98)	< 0.001
Weight‐for‐age z‐score	−0.96 (1.25)	−1.19 (1.37)	−0.59 (0.89)	< 0.001
Weight‐for‐length z‐score	0.02 (1.20)	‐0.05 (1.28)	0.13 (1.03)	0.306
Stunted, *n* (%)	102 (21.6)	79 (27.2)	23 (12.6)	< 0.001
Wasted, *n* (%)	22 (4.7)	20 (6.9)	2 (1.1)	0.004
Underweight, *n* (%)	83 (17.6)	71 (24.4)	12 (6.6)	< 0.001

Abbreviations: ANC, antenatal care; BMI, body mass index; MDD‐W, minimum dietary diversity for women; MUAC, mid‐upper arm circumference; SES, socioeconomic status.

^a^ Data shown if either mother or child has thiamine biomarker and/or human milk thiamine concentration. Sample size for different outcomes may vary. Values are mean (SD) unless otherwise indicated.

^b^ Merchant, business or government employee.

^c^ Self‐reported indicators of socioeconomic status (SES) included education and occupation of the mother and household head, household size and composition, housing characteristics, access to utilities and household ownership of assets and land. These proxy indicators were used to estimate household SES index using principal component analysis.

^d^ Consumption of ≥ 5 out of 10 defined food groups in the previous 24 h/day before going to hospital was considered as meeting the MDD‐W (FAO 2021).

^e^ Heel and toe walk test were considered abnormal if participant was unable to walk on either heels or toes for 10 m. Total sample size for heel and toe walk and rise from squat: *n* = 136 (hospital *n* = 87; community *n* = 49).

^f^ Breastfeeding with certain liquids (water, water‐based drinks, fruit juice) (WHO 2008).

^g^ Breastfeeding with formula and/or animal milk (WHO 2021).

^h^ Breastfeeding with soft foods or other combinations (WHO 2021).

### 3.2 Maternal Thiamine Status and Associations With Human Milk Thiamine Content

Among all women combined, the prevalence of ThDP < 95 nmol/L was 78.5%, elevated ETKac (> 1.25) was 52.6%, and low MTh < 90 µg/L was 45.4% (Table 2). While ThDP did not differ between the hospital and the community group, more women had elevated ETKac (indicating poorer thiamine status) (57.5% vs. 44.3%, *p* = 0.005) and low MTh (54.1% vs. 31.3%, *p* < 0.001) in the hospital compared to the community group. Mean MTh was 82.1 (43.2, 135.5) µg/L in the hospital cohort and 114.6 (80.1, 150.6) µg/L in the community comparison group. Among all women combined, there was a weak Spearman correlation with continuous infant age (coefficient *ρ* = 0.11, 0 = 0.017) with slightly lower MTh concentration in early lactation. This was not significantly different when the duration of lactation was categorized by month (Supporting Information S1: Figure 1).

**Table 2:** Thiamine status among women and their breastfed infants < 6 months of age in the hospital and community comparison group of the Lao Thiamine Study.

	Infants			Women		
Characteristics	Hospital	Community	*p*	Hospital	Community	*p*
*N* a	192	167		306	183	
Whole blood ThDP^b^ (nmol/L)	64.1 (40.0, 91.8)	68.3 (49.8, 87.9)	0.439	71.2 (54.4, 88.6)	75.9 (58.4, 95.2)	0.153
Low ThDP (< 95 nmol/L), *n* (%)	148 (77.5)	134 (81.2)	0.388	246 (80.7)	137 (74.9)	0.132
ETKac	1.27 (1.13, 1.57)	1.22 (1.13, 1.38)	0.041	1.28 (1.16, 1.47)	1.24 (1.15, 1.32)	< 0.001
Elevated ETKac (> 1.25), *n* (%)	86 (52.8)	62 (47.0)	0.323	176 (57.5)	81 (44.3)	0.005
Human milk total thiamine (μg/L)	—	—	—	82.1 (43.2, 135.5)	114.6 (80.1, 150.6)	< 0.001
Human milk total thiamine < 90 μg/L, *n* (%)	—	—	—	159 (54.1)	57 (31.3)	< 0.001

*Note:* Results shown as *n* (%), mean (SD) and median (IQR).

Abbreviations: ETKac, erythrocyte transketolase activity coefficient; ThDP, thiamine diphosphate

^a^ Results shown for infants or women who have at least one thiamine biomarker result. Sample size varies by outcome.

^b^ For conversion of whole blood ThDP to µg/L, multiply nmol/L * 0.42531.

**Figure 2:**
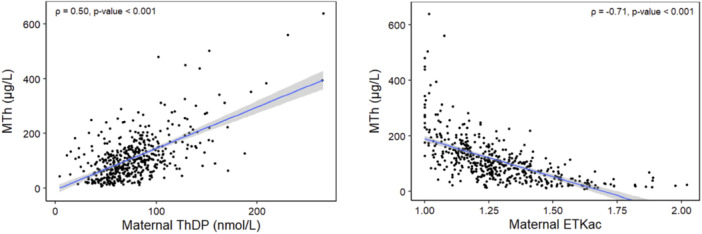
Associations between maternal thiamine status and human milk thiamine concentration.

Maternal thiamine status was significantly associated with MTh concentration (Figure 2). This association was similar in unadjusted and covariate‐adjusted regression models, as well as via Spearman correlation. Specifically, maternal ThDP was moderately correlated with MTh (*ρ* = 0.5) and ETKac was strongly correlated with MTh (*ρ* = −0.71). This correlation did not depend on exclusivity of breastfeeding nor on duration of breastfeeding.

### 3.3 Human Milk Thiamine Content and Associations With Infantile Thiamine Status

**Figure 3:**
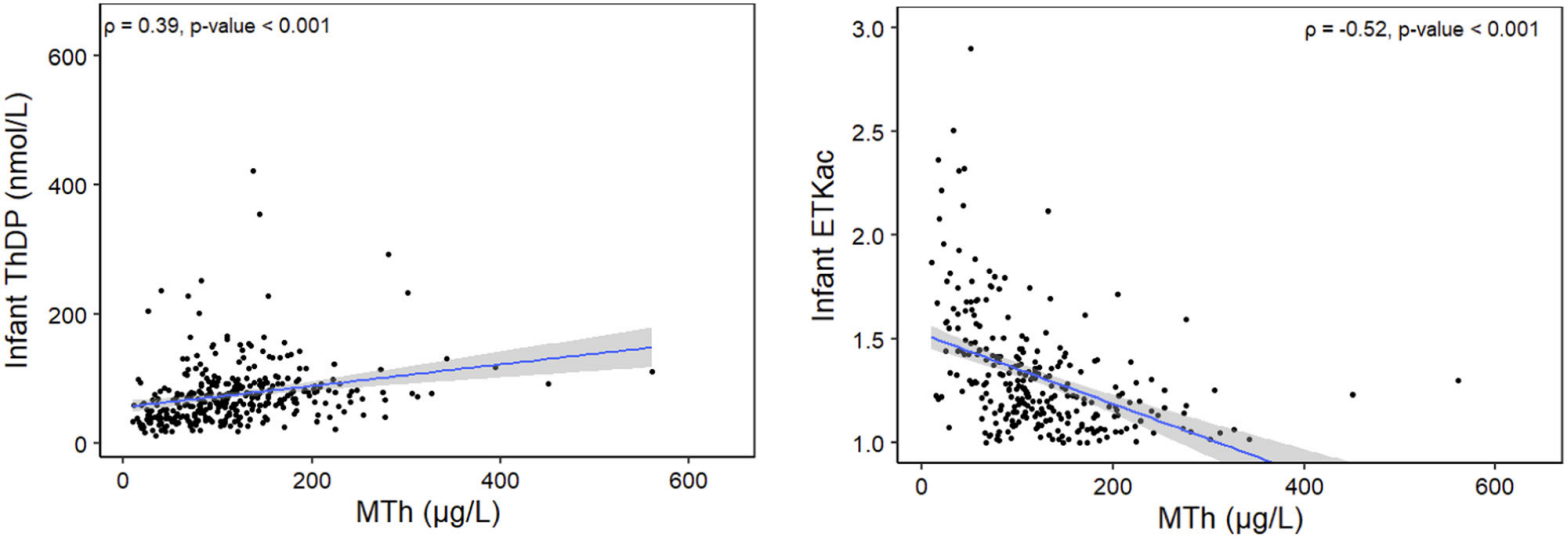
Associations between human milk thiamine concentration and infant thiamine status.

Among all infants combined, the prevalence of ThDP < 95 nmol/L was 79.2% and elevated ETKac (> 1.25) was 50.2% (Table 2), and the prevalence of low ThDP and elevated ETKac did not differ between the hospital and community groups. Although the association between MTh and infant thiamine status was slightly weaker than that between maternal thiamine status and MTh, the association was strongly significant and in the expected direction (Figure 3) in both regression models and when assessed via Spearman correlation. Specifically, MTh was moderately correlated with infant ThDP concentration (*ρ* = 0.39) and with infant ETKac (*ρ* = −0.52). In contrast to maternal status, this correlation did differ by exclusivity of breastfeeding but not by infants' age. Specifically, among exclusively breastfed infants the correlation was stronger for both ThDP (*ρ* = 0.42) and ETKac (*ρ* = −0.58) as compared to non‐exclusively breastfed infants (*ρ* = 0.25 and *ρ* = −0.25).

### 3.4 Cutoff of MTh Associated With Maternal and Infant ETKac > 1.25

MTh was a reliable predictor of a maternal ETKac cutoff of > 1.25 with an AUROC of 0.83 (0.79, 0.87), and with a proposed MTh cutoff of 102 μg/L, a sensitivity of 0.77 and specificity of 0.73 (Table 3). This proposed MTh cutoff predicting elevated maternal ETKac was lower among mothers with infants < 3 months of age (87 μg/L) compared to mothers of infants ages 3–6 months (108 μg/L). Interestingly, the proposed MTh cutoff for maternal ETKac > 1.25 was only 87 μg/L among mothers of hospitalized infants, while it was 115 μg/L among mothers in the community comparison group. Because mothers of hospitalized infants had lower SES status and were more likely food insecure, this difference may in part be explained by poverty‐related factors. Specifically, the proposed MTh cutoff was slightly lower among women in the lowest compared to the highest SES quintile (80 vs. 87 μg/L) and lower among mothers reporting household food insecurity compared to household food security (94 vs. 115 μg/L; Table 3).

**Table 3:** AUROC, potential cut‐off and associated sensitivity and specificity of human milk thiamine concentration to predict elevated ETKac in women and infants.

Outcome	Subsample	*N*	AUROC (95% CI)	Optimal MTh cut‐off (μg/L)	Sensitivity at cut‐off	Specificity at cut‐off
Maternal elevated ETKac	Full sample	476	0.83 (0.79, 0.87)	102.1	0.77	0.73
	Exclusively breastfed	388	0.85 (0.81, 0.89)	101.6	0.78	0.74
	Infant less than 3 months	318	0.83 (0.79, 0.88)	86.9	0.72	0.80
	Infant 3–6 months of age	158	0.84 (0.78, 0.90)	108.1	0.77	0.78
	Mothers of hospitalized infants	294	0.86 (0.81, 0.90)	86.8	0.76	0.80
	Mothers in community	182	0.77 (0.70, 0.84)	114.6	0.79	0.73
	Lowest SES quintile	103	0.80 (0.70, 0.88)	80.3	0.75	0.77
	Highest SES quintile	92	0.85 (0.76, 0.93)	86.9	0.76	0.93
	Food insecure	161	0.84 (0.78, 0.91)	94.4	0.81	0.77
	Food secure	309	0.82 (0.78, 0.87)	114.6	0.85	0.67
Infant elevated ETKac	Full sample	230	0.77 (0.71, 0.83)	109.0	0.71	0.74
	Exclusively breastfed	211	0.77 (0.71, 0.84)	108.9	0.71	0.74
	Infant less than 3 months	147	0.74 (0.66, 0.82)	108.3	0.72	0.69
	Infant 3–6 months of age	83	0.88 (0.80, 0.96)	121.2	0.80	0.88
	Hospitalized infants	124	0.77 (0.68, 0.85)	106.0	0.74	0.67
	Community infants	106	0.79 (0.71, 0.88)	119.4	0.77	0.76
	Lowest SES Quintile	45	0.79 (0.64, 0.94)	93.0	0.68	0.82
	Highest SES Quintile	42	0.83 (0.69, 0.96)	119.4	0.91	0.75
	Food insecure	73	0.78 (0.67, 0.90)	109.5	0.83	0.64
	Food secure	155	0.76 (0.68, 0.84)	119.4	0.74	0.69

Abbreviations: AUROC, area under the receiver operating characteristic; ETKac, erythrocyte transketolase activity coefficient; MTh, human milk thiamine; SES, socioeconomic status.

Among all exclusively and predominantly breastfed infants combined, MTh was strongly predictive of infant ETKac cutoff of > 1.25 with an AUROC of 0.77 (0.71, 0.73) with a proposed MTh cutoff of 109 μg/L, and a sensitivity of 0.71 and a specificity 0.74. This proposed cutoff was almost identical when the analysis was limited to exclusively breastfed infants (Table 3). However, similar to the findings among women, we found that the proposed cutoff for elevated infant ETKac was lower among hospitalized infants (106 μg/L) compared to the community comparison group (119 μg/L). The proposed MTh cutoff was also lower among younger infants compared to older infants (108 vs. 121 μg/L), among infants in the lowest SES quintile compared to the highest SES quintile (93 vs. 119 μg/L), and among infants living in food insecure households compared to food secure households (110 vs. 119 μg/L), respectively.

**Figure 4:**
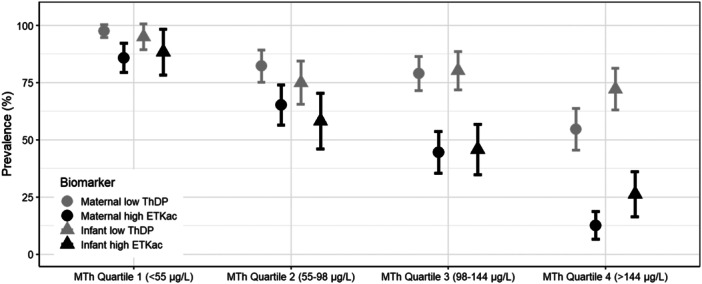
Prevalence of women and their infants with high erythrocyte transketolase activity coefficient and low whole blood thiamine diphosphate concentration by quartile of human milk thiamine concentration^1^. ^1^ High ETKac defined as ETKac > 1.25; low ThDP defined as ThDP < 95 nmol/L.

We also explored how the prevalence of low thiamine status differed by MTh quartile (Figure 4). The vast majority of both women and infants had low ThDP and elevated ETKac in the lowest MTh quartile (< 55 μg/L), and the prevalence of elevated ETKac was 12.6% among women and 26.3% among infants in the highest MTh quartile (> 144 μg/L). The prevalence differed by quartile for low ThDP in women (*p* < 0.001) and for elevated ETKac in both women and children (*p* < 0.001).

## 4 Discussion

The present study found that MTh was moderately to strongly correlated with maternal thiamine status and that infant thiamine status was moderately correlated with MTh among lactating women and their breastfed infants < 6 months of age in northern Lao PDR. Together these results confirm previous reports that maternal thiamine deficiency puts breastfed infants at risk of thiamine deficiency (World Health Organization 1999). It is important to review these findings considering the overwhelming evidence for the overall benefits of exclusive breastfeeding in the first 6 months of life followed by continued breastfeeding up to 2 years of age (Victora et al. 2016). Thus, considering that maternal thiamine supplementation increases thiamine concentration in human milk (Allen 2012), and in view of the severe risks of clinical manifestations and adverse long‐term effects of thiamine deficiency among infants (Smith et al. 2021), increasing maternal thiamine status either through supplementation and/or dietary diversification is urgently needed to ensure both the women's and the young infants' health and wellbeing. As previously reported, women and infants and young children in this study were deficient in multiple micronutrients in addition to thiamine deficiency (Hess et al. 2024), thus supplementation with multiple micronutrients during pregnancy and lactation may be most beneficial.

The correlation between maternal thiamine status and MTh among Laotian women, was similar to associations found between maternal ThDP concentration and MTh in a refugee population on the Thai Myanmar border (Stuetz et al. 2012). Moreover, other studies found that MTh depended on dietary thiamine intake during pregnancy and lactation (Ortega et al. 2004; Kodentsova and Vrzhesinskaya 2006), although this was not the case in all studies (Daniels et al. 2019). While most thiamine supplementation trials used high‐level, therapeutic doses (Dror and Allen 2018), a recent dose–response trial among Cambodian women found that a low dose of 1.2 mg supplementary thiamine for 24 weeks increased thiamine concentration in human milk to levels found in regions without beriberi (Gallant et al. 2021). In contrast, the latter study found a significant impact on infant's ETKac only in the group receiving the highest supplementary dose of 10 mg thiamine compared to placebo (Gallant et al. 2021). In the present study, we found a correlation between MTh and infants' ThDP and ETKac, although this association was not as strong as the one between maternal thiamine status and MTh.

We found that a MTh cutoff < 102 µg/L predicted maternal elevated ETKac among all women combined and MTh < 109 µg/L among all exclusively and predominantly breastfed infants combined, respectively. This was surprising because among well‐nourished, unsupplemented women in Bangladesh, Brazil, Denmark, and the Gambia participating in the MILQ study, the 50th percentile of thiamine concentration in human milk was ≈114 μg/L at 3–4.5 months postpartum and 82 μg/L in the first month (personal communication, Lindsay Allen). Even though our values for milk thiamine concentration were higher than those in the MILQ study, we found a similar difference in the proposed MTh cutoff by infant age group, with lower MTh during the early months of an infant's life. When using the AUROC framework in SES subgroups, we found that the proposed cutoff for both elevated maternal and infant ETKac > 1.25 was lower among the lowest compared to the highest SES quintile and those living in food insecure and compared to food secure households, respectively. Thus, our study suggests that in more vulnerable groups, the selected cutoff for identifying women and infants with elevated ETKac is lower than comparably less vulnerable groups, which may in part explain the difference between the present study and those in the MILQ study. The reason for this difference is uncertain and is likely due to the complex interplay between poverty and nutritional and health status. Moreover, human milk is a complex biological system that not only depends on many maternal factors, there also seems to be some bidirectional interaction from the infant to the mother (Christian et al. 2021).

Although the prevalence of low ThDP did not differ between women in the hospital and the community comparison group, more women in the hospital cohort had elevated ETKac indicating a higher risk of thiamine deficiency. This was expected because women were eligible for enrollment in the hospital cohort of the Lao Thiamine study because their infants were admitted to the hospital for clinical signs and symptoms suggestive of thiamine deficiency, while mother–infant dyads in the community comparison group were frequency matched based on the child's age, sex and location of residence. Moreover, more women in the hospital cohort reported feeling lethargic, having reduced appetite and experiencing tingling in fingers and toes in the previous 2 weeks, suggesting the women in the hospital cohort were not only of poorer socioeconomic status and experienced food insecurity, but also suffered health concerns themselves, of which some may likely have been due to severe thiamine deficiency. The Lao National Nutrition Strategy to 2025 and Plan of Action 2016–2020 states that there is currently inadequate scientific evidence to mandate the provision of thiamine supplementation during pregnancy (Government of Lao PDR 2015). However, the distribution of thiamine supplements to pregnant women in the second and third trimesters was recommended in high‐risk areas during health center staff training, in addition to iron‐folic acid supplements. Despite almost 80% of interviewed health centers in the study area reported distributing antenatal high‐dose thiamine supplements, only approximately a third of women reported taking thiamine supplements during the pregnancy with the study child (Smith, Sitthideth, et al. 2022).

It is important to note that even in the quartile with the highest thiamine concentration in milk, over a quarter of infants had elevated ETKac (> 1.25). ETKac is a functional indicator of thiamine status and this cutoff is generally used to identify high risk of thiamine deficiency (Whitfield 2025). The prevalence of ThDP < 95 nmol/L among infants in the highest MTh quartile was even higher. However, there is no consensus on the appropriate ThDP cutoff to define thiamine deficiency (Whitfield 2025), and when drafting the statistical analysis plan before data analysis, we decided to use a cutoff suggested for adults (Schrijver et al. 1982; Ihara et al. 2005). As previously reported from the Lao Thiamine Study, we found that a cutoff of 64 nmol/L was strongly predictive of ETKac > 1.25 with an AUROC of 0.86 (95% CI: 0.83, 0.90) among infants and young children in the Lao Thiamine Study (Hess et al. 2024). Thus, the cutoff of < 95 nmol/L likely overestimated the prevalence of low ThDP. Nevertheless, even when using the cutoff of < 64 nmol/L, 33.9% infants of mothers in the highest MTh quartile would be defined as thiamine deficient in the present study.

One limitation of the present study is that the participants were not representative of the study area. Moreover, infants in the hospital cohort were enrolled for cardiovascular, respiratory, neurological, and/or behavioral disorders suggestive of thiamine deficiency, and a relatively large number of children had to be excluded because hospitalized infants with life‐threatening conditions received thiamine administration before blood draw as part of their medical treatment, which was important for ethical reasons (Hess et al. 2024). Thus, we may have excluded more severely thiamine‐deficient infants. Nevertheless, this is a large data set derived from rigorous data collection and robust laboratory analyses, and the enrollment of mother–infant dyads allowed to provide insights into associations between maternal thiamine status, thiamine content in milk and infant thiamine status, information which very few studies have published to date.

In conclusion, maternal thiamine status predicts the thiamine concentration in human milk, and thiamine status of breastfed infants < 6 months of age depends on the thiamine provided through this milk. It may be interesting to explore the usefulness of MTh as a biomarker of thiamine status among women and their breastfed infants in population‐representative surveys. Considering the recognized importance of breastfeeding, and the adverse effects of thiamine deficiency for the young child, maternal thiamine supplementation, ideally as part of a multiple micronutrient supplement, and/or increasing dietary diversity is urgently needed to improve thiamine status among these women and their infants.

## Author Contributions

S.Y.H. and C.D.A. conceived the present study protocol; S.Y.H. and T.J.S. developed the data collection questionnaires; S.K. translated all data collection questionnaires to Lao language; T.J.S., D.S., S.K., and S.Y.H. planned the local study implementation; T.J.S. and D.S. supervised the data collection; D.H., K.S.J., D.A.P., S.R.M., and L.H.A. performed laboratory analyses; C.D.A. performed statistical analyses; S.Y.H. drafted the manuscript. All authors critically reviewed the draft manuscript and read and approved the final manuscript.

## Conflicts of Interest

The spouse of S.Y.H. previously worked and currently consults for the Bill & Melinda Gates Foundation. All other authors declare no conflicts of interest.

## Supporting information

Supporting information.

## Data Availability

The data that support the findings of this study are openly available in Open Science Framework at https://osf.io/jfke3, reference number DOI 10.17605/OSF. IO/JFKE3.
