# Supplementary material for: Thiamine Concentration in Human Milk Is Correlated With Maternal and Infant Thiamine Status: A Cross‐Sectional Analysis of the Lao Thiamine Study
Source: Matern Child Nutr. 2025 Apr 10;21(3):e70027. doi: 10.1111/mcn.70027 (PMC12150126; doi:10.1111/mcn.70027)
Supplement: Supplementary file 1 — Supporting information. [file MCN-21-e70027-s001.docx]

**Online supplementary material**

**Thiamine concentration in human milk is correlated with maternal and infant thiamine status: a cross-sectional analysis of the Lao Thiamine Study**

Page

| **Supplementary** **Table S1**: Eligibility criteria for study participants in the Lao Thiamine Study | 2 |
| --- | --- |
| **Supplementary Figure S1**: Human milk thiamine concentration by infant age | 4 |
|  |  |
|  |  |

**Supplementary** **Table S1**: Eligibility criteria for study participants in the Lao Thiamine Study

| Cohort and study participant | Target age range | Inclusion criteria | Exclusion criteria |
| --- | --- | --- | --- |
| Hospital  children | 21 days to  <18 months | - Liver enlargement (>2cm below right costal margin on supine exam while relaxed) - Edema - Tachypnea (> 60/min for 3-8 wks; > 50/min for 2-11 mo; > 40/min for 12-18 mo) - Tachycardia (heart rate >160/min for <12 mo; >120/min for 12-18 mo) - Oxygen saturation < 92% - Difficulty breathing (i.e. chest in-drawing, nasal flaring) - Refusal to breastfeed or refusal of infant formula or food for greater than 24 hours - Repetitive or recurring vomiting with no obvious other cause (i.e. vomiting >3 times in past 24 hours) - Persistent crying not relieved by soothing and feeding with no obvious other cause - Hoarse voice/cry or loss of voice - Nystagmus or other unusual eye movements - Muscle twitching - Loss of consciousness - Convulsion - Opisthotonus / abnormal posturing - Acute paralysis / flaccid paralysis   and Informed written consent by at least one parent or the primary caregiver | n/a |
| Hospital mothers | None | - Child enrolled in hospital cohort  - Informed written consent | - Severe acute illness warranting immediate hospital referral  - Unable to provide informed consent due to reduced decision-making ability |
| Community children | 21 days to  <18 months | - Meeting frequency matched characters (age, sex and location of residence) based on hospital cohort  - Informed written consent by at least one parent or the primary caregiver | - Severe acute illness warranting immediate hospital referral |
| Community mothers | None | - Child enrolled in community cohort  - Informed written consent | - Severe acute illness warranting immediate hospital referral  - Unable to provide informed consent due to reduced decision-making ability |

**Supplementary figure S1:** Human milk thiamine concentration by infant age^1^

**
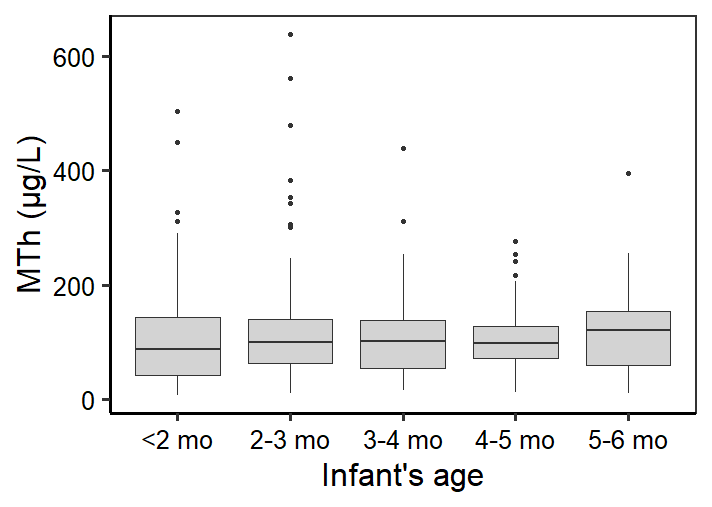
**

**^1^**Distribution by infant age category not statistically different. Kruskal-Wallis p-value=0.296.
